# Prevalence of Epilepsy in Children With Autism Spectrum Disorder Referred to the Autism Clinic in a Tertiary Care Hospital in Bangladesh

**DOI:** 10.7759/cureus.103617

**Published:** 2026-02-14

**Authors:** Mohammad Monir Hossain, Ayesha Siddika, Narayan Saha, Muhammad M Rahman, Ariful Islam, Yamin Shahriar Chowdhury, Kanij Fatema, Bithi Debnath

**Affiliations:** 1 Pediatric Neurology, National Institute of Neurosciences and Hospital, Dhaka, BGD; 2 Reproductive and Child Health, National Institute of Preventive and Social Medicine (NIPSOM), Dhaka, BGD; 3 Pediatric Neurology, Institute of Pediatric Neurodisorder and Autism (IPNA) Bangabandhu Sheikh Mujib Medical University, Dhaka, BGD

**Keywords:** autism, comorbid, eeg, epilepsy, seizure

## Abstract

Background: Autism spectrum disorder (ASD) is a neurodevelopmental disorder believed to be strongly associated with epilepsy. The prevalence of epilepsy among children with ASD ranges widely, and an increased prevalence is observed in low-resource countries. Besides, individuals with ASD have an increased chance of abnormal epileptiform activity on EEG, irrespective of the presence of epilepsy. There is a lack of understanding of their intricate relationship and possible associations. Hence, we conducted this study to evaluate the prevalence and types of epilepsy and its association with abnormal EEG findings in children with ASD.

Materials and methods: This cross-sectional observational study was conducted at the autism clinic in the Department of Pediatric Neurology at the National Institute of Neurosciences and Hospital, Dhaka, from July 2020 to December 2020. Following informed written consent from parents or legal guardians, 100 children with ASD aged 1-17 years were included in this study, irrespective of sex, race, or ethnicity, after meeting the inclusion and exclusion criteria. Ethical clearance was obtained from the Bangladesh Medical Research Council before the commencement of the study. The Diagnostic and Statistical Manual of Mental Disorders, Fifth Edition (DSM-5), was used to confirm the diagnosis of ASD. Then, data were collected using a structured questionnaire. EEG was performed on each study patient using a digital EEG machine. After collection, the data were analyzed using SPSS Statistics version 24.0 (IBM Corp. Released 2016. IBM SPSS Statistics for Windows, Version 24.0. Armonk, NY: IBM Corp.).

Results: The mean age of the participants was 4.47 ± 2.35 years, with a male predominance. Among them, 20 (20%) patients had epilepsy. Out of these children having epilepsy, 12 (12%) had a focal seizure, and the rest had other types of seizures. The average age at seizure onset was 25.14 ± 26.09 months. Among comorbidities, hyperactivity was at the top, followed by sleep disturbance, intellectual disability, and attention-deficit/hyperactivity disorder. EEG revealed that 62 (62%) patients had abnormal electrographic changes, where focal epileptiform discharges were documented in 35 (35%) patients. Additionally, 42 (42%) children with abnormal EEG findings had no clinical seizure. A significant association was found between clinical seizures and EEG abnormalities (p < 0.05). ASD children with epilepsy showed a significant association (p < 0.05) with hyperactivity in this study.

Conclusions: We found that one-fifth of children with ASD had clinical epilepsy, where the focal seizure was the predominant seizure type. Although EEG abnormalities were found in two-thirds of patients with ASD, there were many cases in which seizures were not detected clinically. A significant association was found between clinical seizures and EEG abnormalities. Further analysis could deepen the understanding of this finding and its implications.

## Introduction

​​​Autism spectrum disorder (ASD) has become a major public health concern due to its increasing global prevalence. It is a neurodevelopmental disorder characterized by challenges in social reciprocity and communication, along with restricted, repetitive, and stereotyped behaviors and interests [1]. Worldwide, about 1 in 160 children have been identified with ASD, which varies with geographical distribution [2]. In South Asia, the current pooled prevalence of ASD is 0.31% and 0.15-8% in Bangladesh, with a higher number in urban areas of the country [3]. This wide variation in prevalence is likely due to differences in study inclusion criteria and sites. Dhaka City, the capital of Bangladesh, has the highest prevalence of ASD among the other cities of the country. However, these numbers are likely underreported, as it has become a significant health problem in the country [4]. ASD and other neurodevelopmental disorders in children impose a significant burden on individuals, families, and societies worldwide. These disorders contribute to profound disability, increased mortality, and diminished quality of life throughout a child’s lifespan. It also impacts financially. The lifetime cost of autism alone is estimated to be 2 million USD per person [4,5].

ASD individuals struggle with co-occurring medical conditions more frequently than the general population, including attention deficit, language disorder, sleeping problems, intellectual disability (ID), and aggression. These patients also have a higher prevalence and increased risk for epilepsy, which is a chronic condition characterized by the abnormal, excessive hypersynchronous firing of cortical neurons, causing unprovoked seizures [6]. A recent systematic review reported that epilepsy can co-exist in 7% of autistic children, with higher prevalence in adolescents and preschool children. In comparison, earlier studies estimated a broader range of 5-46% in autistic children [7-9]. The high occurrence of epilepsy in individuals with ASD suggests a shared underlying pathophysiology [10], potentially due to an imbalance in excitatory or inhibitory neurotransmitters leading to over-connectivity in certain parts of the brain. However, multiple genetic and environmental factors also play a role in both ASD and epilepsy, so the exact pathophysiology remains unclear [11].

Additionally, individuals with ASD also have a 15-85.8% higher likelihood of showing abnormal epileptiform activity, including epileptiform discharge on EEGs with or without seizures [12-13]. The frequency and location of their interictal spikes also differ from those of healthy children [11]. Moreover, the diagnosis of epilepsy is difficult in individuals with ASD due to behavioral abnormalities associated with focal seizures with impaired awareness and absence seizures, which could be due to autism as well [14]. This intricate relationship between epilepsy and ASD underscores the importance of early detection and intervention, as well as the need for ongoing research to correlate ASD, epilepsy, and abnormal EEG activities to alleviate its burden.

The burden of ASD and epilepsy is particularly pronounced in resource-limited settings. A higher prevalence of concurring ASD and epilepsy is often observed in low Human Development Index (HDI) countries [15]. Bangladesh is one of the low HDI countries that has very little data regarding the double burden of ASD and epilepsy in children. Evaluating the prevalence of co-occurrence of epilepsy and its types in children with ASD and its association with EEG abnormalities can serve as a foundation for future studies. The study can deepen our understanding of the association and impact of epilepsy in this population and may help to design an effective management strategy.

## Materials and methods

Study design and population

This was a cross-sectional observational study conducted at the Autism Clinic, Department of Pediatric Neurology, National Institute of Neurosciences and Hospital (NINS&H), a tertiary hospital in Dhaka, Bangladesh. A total of 100 children with ASD aged 1 to 17 years who attended the clinic during the six-month study period from July 2020 to December 2020 were recruited. ASD was diagnosed according to the Diagnostic and Statistical Manual of Mental Disorders, Fifth Edition (DSM-5) criteria by a child psychologist. We excluded cases such as cerebral palsy, microcephaly, dysmorphism, or laboratory evidence of congenital infection or neurometabolic disorders. The researcher developed a structured questionnaire to emphasize the study's purpose. It included sociodemographic variables such as age, sex, residence, the educational status of the child and parents, monthly family income, and other related information. It also contained some relevant cultural variables like beliefs and attitudes of parents regarding their child’s disease and some variables related to epilepsy, such as duration of illness, age of onset, type of epilepsy (according to seizure origin), seizure frequency, family history of epilepsy, antiepileptic therapy at the time of assessment, EEG, and imaging findings.

Study procedure

Formal ethical approval was obtained from the Bangladesh Medical Research Council (BMRC). Children with ASD who met the inclusion and exclusion criteria were selected as study participants. Informed written consent was obtained from all parents or caregivers of study patients. Each parent/caregiver was interviewed face-to-face using a structured questionnaire to collect data on the variables. Details regarding the demographic profile and seizure pattern were taken. Children were assessed for attention-deficit/hyperactivity disorder (ADHD) using the Conners' Parent Rating Scale, completed by the parents, which is a popular research and clinical tool for obtaining parental reports of childhood behavior problems. The ADHD symptoms must be present in at least two settings, and they must interfere with the child's function and development. If the only behavior was hyperactivity and occurred only in specific situations, the child was categorized as having only hyperactivity. IQ was tested using the Wechsler Preschool and Primary Scale of Intelligence by a pediatric psychologist. These validated psychometric tools were used with prior permission of the institute.

Each study patient went through an EEG on a digital EEG machine. A 21-channel digital EEG was performed using a Nihon Kohden EEG machine (model 1200), manufactured in Japan in 2015. The 10-20 international system of electrode placement was used, and both bipolar and referential montages were monitored. No sedation was administered. However, oral melatonin was administered at 0.2 mg/kg/dose to children with difficulty sleeping during the EEG procedure. The EEG was recorded in both sleep and wake states. The average recording time was 40 minutes. Parents or caregivers were asked to keep children awake for approximately 6 hours the night before the recording. All EEGs were interpreted, reviewed, and reported by a pediatric neurologist. The epilepsies were classified according to the International League Against Epilepsy 2017 classification of seizure types [16]. MRI was advised for children having a focal seizure, abnormal head shape, or focal neurological signs.

Statistical methods

Data were collected using a pre-designed data collection sheet. The statistical analysis was conducted using SPSS Statistics version 24.0 (IBM Corp. Released 2016. IBM SPSS Statistics for Windows, Version 24.0. Armonk, NY: IBM Corp.). Qualitative variables were reported as frequencies and percentages, and quantitative variables as means ± standard deviations. A significance test was performed using the chi-square test. The level of significance was set at p < 0.05.

Ethical implications

Before initiation of the study, the protocol was reviewed, and formal approval was obtained from BMRC (BMRC/Revenue-Grant/2019-2020/753/1-31). All methods were performed in accordance with the Declaration of Helsinki. Ethical measures were implemented throughout the study period to maintain high standards of participant confidentiality and anonymity. Informed written consent was taken from the parents or guardians.

## Results

Demographic characteristics

A total of 100 children with ASD were enrolled in this study. The sociodemographic profile of the participants is discussed in Table 1. The mean age of participants was 4.47 ± 2.35 years (range: 1-15), with male predominance. About 46 (46%) children resided in urban areas, and 63 (63%) belonged to the middle class. In this study, 83 (83%) had never attended school. The parents of the study participants are mostly literate. They could read, write, and calculate for effective community functioning.

**Table 1:** Sociodemographic profile of study participants (n = 100) SD: standard deviation

Variables	Number (%)
Age (in years) (mean ± SD)	4.47 ± 2.35
Sex	
Male	76 (76)
Female	24 (24)
Residence	
Rural	32 (32)
Urban	46 (46)
Semi-urban	22 (22)
Educational status of children	
Never went to school	83 (83)
Went to a special school	15 (15)
Conventional normal school	2 (2)
Education of father	
Literate	100 (100)
Illiterate	0 (0)
Education of mother	
Literate	99 (99)
Illiterate	1 (1)
Occupation of father	
Service holder	67 (67)
Business	32 (32)
Farmer	1 (1)
Occupation of mother	
Service holder	13 (13)
Housewife	87 (87)
Socioeconomic status	
Lower	8 (8)
Middle	63 (63)
Higher	29 (29)

Risk factors

Of all participants, 10 (10%) children had a family history of ASD, and 9 (9%) had a history of consanguineous parents. The majority (74%) were delivered at term via lower uterine cesarean section (LUCS). The mean birth weight was 2.81 ± 0.60 kg (range: 1.40-4.30). Birth history showed that 25 (25%) were given oxygen for breathing, eight (8%) were treated with antibiotic(s) for infection, 14 (14%) had neonatal jaundice, and seven (7%) had neonatal seizures. Approximately 12 (12%) required specialized care in the NICU. Detailed information is shown in Table 2.

**Table 2:** Risk factors among study participants (n = 100) * multiple response ASD: autism spectrum disorder, NVD: normal vaginal delivery, LUCS: lower uterine ceaserian section, DM: diabetes mellitus, NICU: neonatal intensive care unit

Risk factors*	Number (%)
Family history of ASD	10 (10)
Family history of neurodevelopmental disorder	4 (4)
History of consanguineous parents	9 (9)
Mother drug history	2 (2)
Mother Pre-eclampsia	6 (6)
Mother eclampsia/toxemia	1 (1)
Mother DM	3 (3)
Infection	6 (6)
Mode of delivery	
NVD	26 (26)
LUCS	74 (74)
Birth history	
Term	78 (78)
Pre term	22 (22)
Mean birth weight (in kg) (range)	2.81 ± 0.60 (1.40-4.30)
Required oxygen support	25 (25)
Special care NICU	12 (12)
Infection care with antibiotics	8 (8)
Neonatal seizure	7 (7)
Neonatal jaundice	14 (14)

Neurodevelopmental status

The neurodevelopmental status of the children is shown in Table 3. The average loss of acquired skill of the study participants was 24.24 ± 8.59 months (range: 12-72). The majority, 89 (89%), had delayed/absent telling of their full name; 86 (86%) had spontaneous sentences; 66 (66%) had spontaneous phrases; 61 (61%) had delayed polysyllabic consonant babbling; and 60 (60%) had observed delayed development of social smile. In contrast, the timely development of body gesture and vocalization was observed in 74 (74%) and 55 (55%), respectively.

**Table 3:** Neurodevelopmental status of study participants (n = 100) SD: standard deviation

Variables	Number (%)
Loss of acquired skill (month) (mean ± SD) (range)	24.24 ± 8.59 (12-72)
Milestone social smile	
Onset of development (month) (mean ± SD) (range)	3.87 ± 0.92 (2-7)
Delayed	60 (60)
Timely	40 (40)
Vocalization	
Onset of development (month) (mean ± SD)	6.44 ± 0.95
Delayed	45 (45)
Timely	55 (55)
Polysyllabic consonant babbling	
Onset of development (month) (mean ± SD) (range)	8.82 ± 1.29 (6-12)
Delayed	61 (61)
Timely	39 (39)
Body gesture	
Onset of development (month) (mean ± SD) (range)	12.51 ± 3.89 (10-48)
Delayed	26 (26)
Timely/present	74 (74)
Spontaneous (not echoed) phrases	
Onset of development (month) (mean ± SD) (range)	20.26 ± 3.77 (12-30)
Delayed	42 (42)
Present/timely	34 (34)
Absent	24 (24)
Sentences	
Onset of development (month) (mean ± SD) (range)	28.17 ± 6.08 (22-48)
Delayed	16 (16)
Present/timely	14 (14)
Absent	70 (70)
Tells full name	
Onset of development (month) (mean ± SD) (range)	34.77 ± 2.24 (30-48)
Delayed	2 (2)
Present/timely	11 (11)
Absent	87 (87)

Comorbidities

Among all comorbidities, hyperactivity was present in 49 (49%) children. Sleep disturbance, ID, and ADHD were identified in 23 (23%), 22 (22%), and 13 (13%) children, respectively.

**Table 4:** Comorbidities among study participants (n = 100) * multiple response ID: intellectual disability, ADHD: attention-deficit/hyperactivity disorder

Comorbidities*	Number (%)
Hyperactivity	49 (49)
Sleep disturbance	23 (23)
ID	22 (22)
ADHD	13 (13)

Prevalence of epilepsy and its types

The prevalence of epilepsy in the studied children with ASD is shown in Table 5. Of all children, 20 (20%) had clinical epilepsy, wherein 12 (12%) had focal epilepsy. About three (3%) were found to have developmental and epileptic encephalopathies, two (2%) had generalized epilepsy, and the remaining three (3%) had combined generalized and focal epilepsy (tonic, myoclonic, and epileptic spasm). The average age at seizure onset was 25.14 ± 26.09 months (range: 2-130). Among children having focal epilepsy, five (5%) had focal onset seizures with impaired awareness, and two (2%) had focal seizures without impaired awareness. Parents of five (5%) were unable to confirm the awareness state at seizure onset.

**Table 5:** Pattern of epilepsy among study participants (n = 100) SD: standard deviation

Epilepsy types	Number (%)
Epilepsy present	20 (20)
Generalized	2 (2)
Focal	12 (12)
Developmental and epileptic encephalopathies	3 (3)
Combined generalized and focal (tonic, myoclonic, and epileptic spasm)	3 (3)
Age of onset (month) (mean ± SD) (range)	25.14 ± 26.09 (2-130)

EEG characteristics

Of all patients, 62 (62%) had abnormal EEG findings (Table 6). Among the abnormal EEG cases, 35 (35%) had focal epileptiform discharges. In contrast, background abnormalities without epileptic discharges were seen in nine (9%), generalized epileptiform discharges in eight (8%), epileptic encephalopathy in seven (7%), and other types of EEG abnormalities (hypsarrhythmia and SWAS) in three (3%).

**Table 6:** EEG characteristics of ASD patients (n = 100) EEG: electroencephalogram, ASD: autism spectrum disorder, SWAS: spike wave activation in sleep

EEG characteristics	Number (%)
Normal	38 (38)
Abnormal	62 (62)
Focal epileptic discharge	35 (35)
Background abnormalities without epileptic discharge	9 (9)
Generalized epileptic discharge	8 (8)
Epileptic encephalopathy	7 (7)
Others (hypsarrythmia and SWAS)	3 (3)

All the patients with clinical seizures had EEG abnormalities. Additionally, EEG was abnormal in 42 (42%) without a history of seizures. Focal epileptiform discharges were the predominant EEG abnormality in both groups. Overall, a significant association was found between children with ASD having clinical seizures and EEG abnormalities (p < 0.05). For further information, see Table 7.

**Table 7:** Comparison of EEG changes in children with ASD having clinical seizure or not (n =100) Values were expressed within parentheses as a percentage (%) over the column in total. * Chi-square test was used to determine the p-value. p < 0.05 is considered significant. ** Fisher’s exact test was used to determine the p-value. EEG: electroencephalogram, CSWS: continuous spike-and-wave during sleep, ASD: autism spectrum disorder

EEG changes	Clinical seizure		p-value
-	Present, N =20 (20%)	Absent, N = 80 (80%)	-
Abnormal, 62 (62%)	20 (20)	42 (42)	<0.001*
Normal, 38 (38%)	0 (0)	38 (38)	
Abnormalities	-	-	-
Focal epileptic discharge	13 (13)	22 (22)	<0.001*
Background abnormalities without epileptic discharge	0 (0)	9 (9)	-
Generalized epileptic discharge	2 (2)	6 (6)	-
Epileptic encephalopathy	5 (5)	2 (2)	-
Others (hypsarrythmia and CSWS)	0 (0)	3 (3)	-

Table 8 illustrates the association between epilepsy and comorbidities in ASD patients. Children with epilepsy had a significant association with hyperactivity compared to patients without epilepsy (p < 0.05). However, sleep disturbance, ID, and ADHD had statistically similar distributions between patients with and without clinical seizures (p > 0.05).

**Table 8:** Association between comorbid conditions and clinical seizure among study participants (n = 100) Values were expressed within parentheses as a percentage (%) over the column in total. * Fisher’s exact test was used to determine the p-value. ** A p-value <0.05 was considered significant. ADHD: attention-deficit/hyperactivity disorder, ID: intellectual disability

Comorbidity	Epilepsy		p-value
-	Present, N = 20 (20%)	Absent, N = 80 (80%)	-
Sleep disturbance	-	-	-
Yes	4 (4)	19 (19)	1.00*
No	16 (16)	61 (61)	-
ID	-	-	-
Yes	7 (7)	15 (15)	0.136*
No	13 (13)	65 (65)	-
ADHD	-	-	-
Yes	2 (2)	11 (11)	1.00*
No	18 (18)	69 (69)	-
Hyperactivity	-	-	-
Yes	16 (16)	33 (33)	0.002**
No	4 (4)	47 (47)	-

## Discussion

Recent articles highlight the strong link between ASD and epilepsy, noting that children with autism have a significantly higher risk of developing epilepsy. This is often due to shared genetic and environmental factors rather than one condition directly causing the other. These children also commonly show abnormal epileptic discharges on EEG. Abnormal EEG can exist in individuals with ASD with or without the presence of epilepsy [1,2]. However, the association between ASD and epilepsy remains underexplored, especially in developing countries, where ASD with epilepsy has a higher frequency than in other countries [3]. This may be due to factors like the lack of access to quality healthcare, which can affect both brain development and the management of seizures. This can lead to misdiagnosis or underdiagnosis, making it appear that there is a higher frequency in these regions.

Research also indicates challenges in diagnosing epilepsy in children with autism due to behavioral and communication differences, which may delay proper treatment. Therefore, we aimed to find the prevalence and types of epilepsy and its association with EEG abnormalities in children with ASD in a tertiary autism clinic in Bangladesh.

In our study, we found that one-fifth, or 20 (20%), of the ASD children had clinical epilepsy, wherein two-thirds of them had focal seizures. EEG abnormalities were found in 62 (62%) children of the cohort. The most commonly found abnormality was focal epileptic discharge, followed by background abnormalities without epileptic discharge. All the children with clinical seizures had abnormal EEG changes. Previous studies reported the range of association as 5-46% [4]. However, there are few studies on this topic, particularly in Bangladesh. In some related studies, generalized tonic-clonic seizure was the predominant type of seizure [4-6]. Of the comorbidities, ASD with epilepsy and ASD with abnormal EEG changes were not significantly associated with sleep disturbance, ID, or ADHD. However, new research highlights that the presence of epilepsy, particularly with abnormal EEG results, is correlated with a higher severity of autism symptoms [7].

In this study, 62 (62%) children of the study subjects had abnormal EEG findings, and of them, nine (9%) had background abnormalities without any epileptic discharge. In a similar context, Akhter et al. found epileptic discharges in 51.9% and non-epileptiform discharges in 15.6% of the population [8]. Among the abnormal EEG findings, 35 (35%) children had focal epileptic discharges. Another study found that 43% of the ASD cohort had epileptic discharges with localized spikes in 73% of them, while other changes were multiple spike foci, generalized, etc. [7]. We found a significant association between abnormal epileptic EEG changes and a history of epilepsy in the cohort. Another study also reported a significant relationship between the two [9]. In ASD, seizures and EEG abnormalities could represent underlying cerebral dysfunction independent of any apparent lesions.

Regarding comorbidities, sleep disturbance was found in 23 (23%), ID in 22 (22%), and ADHD in 13 (13%) children. Another related study found that ID was present in 8-48.7% of ASD, depending on the severity of ASD [17]. The prevalence of ADHD in those with ASD has ranged from 14% to 78% [18]. A similar study found that 18% of the children and adolescents with ASD also had a comorbid diagnosis of ADHD [19]. However, our study found no significant association between ASD with or without clinical seizures and comorbidities such as sleep disturbance, ID, or ADHD. This may be due to the small sample size and the absence of a control group, which precludes drawing causal inferences. However, children having both ASD and epilepsy had a significant association with hyperactivity when compared to children having only ASD.

Our study has a few limitations that should be considered. First, the sample size was small, which may limit the generalizability of the findings. Second, there was no control group, and third, it was based on a single referral center; therefore, the cohort's characteristics may not be representative of other settings, and overestimation of disease burden could lead to selection bias. The study is cross-sectional; hence, correlation could not be established. Further large multicenter studies are required to provide more reliable recommendations.

## Conclusions

The study found that about one-fifth of children with ASD had epilepsy, and focal seizure was the predominant seizure type. But this study pointed out that a high proportion of children had epileptic discharges on EEG, even though they did not have clinical evidence of epilepsy. Children with epilepsy were found to be more hyperactive compared to children without epilepsy. Further analysis could deepen the understanding of this finding and its implications.
